# Psychometric Behaviour of the GAD-7 in Medical Students: Structural Stability, Measurement Equivalence and Contextual Sensitivity

**DOI:** 10.3390/healthcare14050563

**Published:** 2026-02-24

**Authors:** Pablo Duran, Ángel Ortega, Nestor Galban, Ivana Vera, Andrea Díaz, Carla Navarro, Rubén Carrasquero, Juan Salazar, Juan Hernández-Lalinde, Valmore Bermúdez, Erika Vásquez-Arteaga, Diego Rivera-Porras

**Affiliations:** 1 Universidad del Zulia, Facultad de Medicina, Centro de Investigación de Enfermedades Endocrinas y Metabólicas, Maracaibo 4001, Venezuela; pabloduran1998@gmail.com (P.D.); angelort94@hotmail.com (Á.O.); nestorag17@gmail.com (N.G.); ivanaa19.iv@gmail.com (I.V.); andreavalentinadiaz@hotmail.com (A.D.); cpnm18@hotmail.com (C.N.); rubendavidxd@gmail.com (R.C.); juanjsv18@hotmail.com (J.S.); 2 Universidad Simón Bolívar, Facultad de Ciencias Básicas y Biomédicas, Cúcuta 540001, Norte de Santander, Colombia; hernandezjuandiego@gmail.com; 3 Universidad Simón Bolívar, Centro de Investigaciones en Ciencias de la Vida, Barranquilla 080001, Atlántico, Colombia; valmore.bermudez@unisimon.edu.co; 4 Universidad Mariana, Facultad de Humanidades y Ciencias Sociales, Pasto 520001, Nariño, Colombia; 5 Universidad de la Costa, Departamento de Productividad e Innovación, Barranquilla 080001, Atlántico, Colombia

**Keywords:** generalized anxiety, GAD-7, medical students, confirmatory factor analysis

## Abstract

Background: Anxiety symptoms among medical students often emerge at the intersection of sustained academic pressure, anticipatory uncertainty and early professional socialisation, complicating their distinction from transient stress responses. Instruments employed in this context are therefore expected to operate consistently across subgroups while preserving conceptual clarity under non-clinical conditions. The Generalized Anxiety Disorder scale (GAD-7), widely adopted as a brief screening measure, has shown variable factorial behaviour across populations, particularly when applied to student cohorts. Materials and methods: Using confirmatory factor analysis with robust weighted least squares estimation, the latent structure of a culturally adapted Spanish version of the GAD-7 was examined in a sample of medical students enrolled across all academic years at a public university. Model performance was evaluated through multiple fit indices suited for ordinal data, alongside estimates of convergent validity based on average variance extracted and reliability assessed via both Cronbach’s α and McDonald’s ω. Measurement invariance across sex was explored through a sequence of increasingly constrained multi-group models. Results: The unidimensional configuration originally proposed for the scale remained statistically coherent, despite minor tensions between absolute and incremental fit indicators commonly reported in comparable university-based samples. Convergent validity estimates suggested that the latent construct accounted for a substantial proportion of item variance, while reliability coefficients fell within the upper range observed internationally. Invariance testing supported comparability at the configurational and scalar levels, although full metric equivalence was less stable. Conclusions: Rather than resolving ongoing debates regarding the internal structure of the GAD-7, these findings situate its psychometric behaviour within the specific demands of medical education, where anxiety-related symptoms may fluctuate between normative adaptation and clinically relevant distress. This positioning invites further examination of how screening instruments perform when anxiety is shaped as much by institutional context as by individual psychopathology.

## 1. Introduction

Medical education is routinely characterised by sustained exposure to evaluative pressure, temporal overload and early professional accountability, conditions under which anxiety-related symptoms often become structurally embedded rather than episodic. Meta-analytic evidence indicates that anxiety symptoms are common among medical students, with pooled prevalence estimates around one third, although reported rates vary by region and assessment instrument [1]. Such figures, while striking, display substantial heterogeneity, raising concerns about the extent to which variability reflects genuine psychological burden as opposed to measurement artefacts.

Generalized anxiety disorder occupies a particularly ambiguous position in this setting. Core features such as persistent worry, cognitive hypervigilance and somatic tension overlap with adaptive responses encouraged within competitive academic environments. Studies conducted across Europe, Asia and the Americas report mean GAD-7 scores among medical students frequently clustering near conventional screening thresholds, often between 7 and 10 points, blurring the boundary between subclinical distress and clinically relevant anxiety [2,3,4]. This proximity to diagnostic cut-offs amplifies the importance of examining how the instrument behaves psychometrically under conditions of elevated but context-dependent symptom endorsement.

The GAD-7 was originally developed within primary care populations, where its unidimensional structure demonstrated acceptable fit and strong diagnostic correspondence with DSM-based interviews [5]. Subsequent validations across clinical and community samples have largely replicated this structure, although university-based studies have yielded less consistent results. Alternative factorial solutions, frequently distinguishing somatic from cognitive–emotional components, have been tested among university students in Asia and the Middle East, with comparative fit differences often modest and sensitive to estimation method [2,6,7]. When ordinal data characteristics are insufficiently addressed, such discrepancies may reflect analytic distortion rather than substantive construct differentiation.

Reliability indices reported for the GAD-7 among student populations are typically high, with Cronbach’s α values ranging from 0.74 to 0.94 across countries, including Germany, Spain, China and South Korea [3,8,9]. While these coefficients are frequently interpreted as evidence of robustness, elevated internal consistency in brief scales may also signal item redundancy or restricted variance, particularly in homogenous academic samples. Such considerations are rarely examined alongside factorial stability, despite their implications for construct representation.

Measurement invariance across sex introduces an additional layer of complexity. Epidemiological data consistently report higher anxiety symptom levels among female medical students, with effect sizes typically in the small-to-moderate range [1]. Meaningful interpretation of these differences presupposes equivalence in item functioning and latent structure. Evidence on GAD-7 invariance remains mixed: scalar invariance has been supported in some university and primary care samples, while metric invariance appears less stable, with ΔSRMR or ΔCFI values exceeding recommended thresholds in several studies [10,11]. These inconsistencies suggest that symptom salience may vary across groups even when overall construct alignment is preserved.

Within Latin American contexts, psychometric evidence on anxiety screening instruments in non-clinical settings is growing but remains uneven, and it is still comparatively scarce for medical-student cohorts analysed with ordinal CFA and invariance frameworks. Recent work has examined latent structure and measurement equivalence in community samples (e.g., Villarreal-Zegarra et al., 2024 [12]) and in university-student datasets across the region (e.g., Franco-Jimenez & Núñez-Magallanes, 2022 [13]; Moreno-Montero et al., 2025 [14]). However, much of the regional medical-student literature continues to prioritise symptom-burden estimation using imported cut-off scores, with limited attention to latent structure or cross-group validity. In medical students, where anxiety may reflect institutional design as much as individual vulnerability, this gap restricts interpretative depth and cross-study comparability.

Framing the GAD-7 within medical education therefore requires attention not only to its statistical adequacy but to the conditions under which its latent construct is activated. Factorial coherence, convergent validity and invariance function less as confirmatory endpoints than as indicators of how the instrument negotiates a setting where anxiety is simultaneously normative, adaptive and potentially pathological. Such an approach leaves open the question of whether conventional screening logic fully captures the phenomenology of anxiety as experienced during professional training.

### Research Question

To what extent does the GAD-7 maintain factorial coherence, convergent validity and measurement invariance across sex when applied to medical students exposed to sustained academic strain?

## 2. Materials and Methods

### 2.1. Study Design and Participants

An instrumental, cross-sectional design was employed to examine the psychometric behaviour of an anxiety screening instrument within a university-based medical training context. This design is commonly adopted in scale evaluation studies where the primary interest lies in latent structure, reliability and measurement equivalence rather than prevalence estimation [15]. Participants were undergraduate medical students enrolled from first to sixth academic year at a public university in Venezuela. The institutional enrolment registry for the corresponding academic period served as the reference population.

Given the psychometric focus and the practical constraints associated with academic schedules and classroom access, a non-probabilistic quota sampling strategy was implemented. Quotas were proportionally defined according to academic year to preserve representation across training stages. Sample size determination followed recommendations based on the subject-to-item ratio for latent variable modelling, typically ranging between 5 and 10 participants per indicator [16]. For a seven-item instrument, a minimum target of 200 participants was established. The final analytic sample consisted of 227 students after data screening procedures.

Eligibility criteria included age ≥18 years, current enrolment in the medical programme and voluntary participation. No exclusions were applied based on symptom severity or academic performance, in line with recommendations for preserving variance when evaluating measurement properties in non-clinical populations [17].

### 2.2. Instrument and Cultural Adaptation

An adapted Spanish version of the Generalized Anxiety Disorder 7-item scale (GAD-7) was administered. The GAD-7 consists of seven ordinal items rated on a four-point Likert scale ranging from 0 (“not at all”) to 3 (“nearly every day”), yielding a total score between 0 and 21 [5]. The version used was based on the validated Spanish adaptation developed by García-Campayo et al., which has demonstrated adequate psychometric performance in primary care and community samples [8].

Minimal lexical adjustments were introduced to ensure semantic clarity and regional linguistic equivalence. Specifically, the introductory instruction was adapted from a formal second-person register to the informal register commonly used in Venezuelan student surveys (e.g., ‘le han molestado’ → ‘te han molestado’) to improve readability; item stems and response anchors otherwise followed the Spanish adaptation by García-Campayo et al. [8]. These modifications were restricted to phrasing and did not alter item content, response format or scoring procedures, consistent with guidelines for cross-cultural adaptation that prioritise conceptual rather than literal equivalence [18]. The instrument was self-administered using a digital format, with standardised written instructions. The final wording used for administration is available from the corresponding authors upon reasonable request. Trained collaborators were available to provide procedural assistance without engaging in item interpretation.

In addition to the GAD-7, a structured form collected sociodemographic and contextual information, including age, sex, academic year and socioeconomic status. Socioeconomic position was assessed using the modified Graffar scale, widely applied in Latin American epidemiological research [19]. Basic anthropometric and clinical measures were recorded following internationally standardised procedures, although these variables were not incorporated into the latent modelling.

### 2.3. Data Preparation and Preliminary Analyses

Data were inspected for completeness, distributional properties and atypical response patterns prior to model estimation. Multivariate normality was assessed using Mardia’s coefficients, while potential multivariate outliers were examined through robust Mahalanobis distance estimates [20]. As expected for ordinal self-report data in student samples, assumptions of multivariate normality were not met.

Given the ordinal scaling of the GAD-7 items, polychoric correlation matrices were used as input for factor analysis, following methodological recommendations indicating that Pearson correlations may underestimate associations among categorical indicators [21]. No imputation procedures were applied, as missing data were minimal and randomly distributed.

### 2.4. Confirmatory Factor Analysis

An exploratory factor analysis was not undertaken because the GAD-7 has an extensively replicated a priori unidimensional structure in both clinical and community samples, and the present study aimed to test the structural stability and measurement invariance of this theoretically specified model under ordinal estimation. In addition, EFA would ideally require an independent calibration sample (or a split-sample approach) to avoid capitalising on chance, which would reduce power for multi-group invariance testing in our cohort. Accordingly, CFA was prioritised as the principal analytic strategy, and alternative reduced-item specifications were treated as sensitivity analyses rather than as substantive model discovery [15,16].

Construct validity was examined through confirmatory factor analysis (CFA), specifying a single latent factor corresponding to generalized anxiety. Model estimation employed the weighted least squares mean and variance adjusted estimator (WLSMV), which has been shown to perform robustly with ordinal indicators and non-normal distributions, particularly in samples of moderate size [22,23].

Model fit was evaluated using multiple complementary indices: the chi-square statistic (χ^2^), the ratio of χ^2^ to degrees of freedom, the root mean square error of approximation (RMSEA), the standardised root mean square residual (SRMR), the comparative fit index (CFI) and the Tucker–Lewis index (TLI). Conventional thresholds were used as reference points (RMSEA < 0.08; SRMR < 0.08; CFI/TLI ≥ 0.95) while acknowledging ongoing debate regarding their rigidity and sensitivity to sample size and model complexity [24,25]. No data-driven post hoc modifications were introduced.

### 2.5. Convergent Validity and Reliability

Convergent validity was evaluated using the average variance extracted (AVE), calculated from standardised factor loadings. AVE values approaching or exceeding 0.50 were interpreted as indicating that the latent construct accounted for a substantial proportion of item variance [26].

Internal consistency was examined using both Cronbach’s alpha (α) and McDonald’s omega (ω). The inclusion of ω was intended to address the limitations of α under violations of tau-equivalence and to provide a reliability estimate aligned with the latent variable framework used in CFA [17]. Reliability coefficients were interpreted using commonly accepted benchmarks, without treating them as definitive indicators of construct quality.

### 2.6. Measurement Invariance

Measurement invariance across sex was assessed through multi-group CFA, following a hierarchical sequence of increasingly constrained models: configural, thresholds, metric, scalar and residual invariance [27]. Configural invariance examined equivalence of factor structure across groups, while subsequent models-imposed equality constraints on factor loadings, item thresholds and residual variances.

Model comparisons prioritised changes in incremental and absolute fit indices rather than chi-square difference tests alone, given their sensitivity to sample size. Differences in ΔCFI ≤ 0.01 and ΔRMSEA ≤ 0.015 were used as practical criteria for invariance retention, in line with recommendations for categorical data models [28]. This approach allowed for evaluation of whether observed group differences could be interpreted at the latent level.

### 2.7. Statistical Software

Data management and descriptive analyses were conducted using SPSS Statistics v24 (IBM Corp., Armonk, NY, USA). Confirmatory and multi-group models were estimated in R v4.3.1 (R Foundation for Statistical Computing, Vienna, Austria) using RStudio v1.1.463 (Posit Software, Boston, MA, USA). Analyses in R employed the lavaan package for CFA and invariance modelling, supported by semTools for additional psychometric indices (e.g., AVE) and *psych* for reliability computations. Emphasis was placed on parameter behaviour and model stability rather than sole reliance on null-hypothesis testing.

### 2.8. Ethical Considerations

This study was approved by the Endocrine and Metabolic Diseases Research Center’s Bioethics Committee (approval No. 201704-015, 25 January 2024; minutes/act No. 202401-025). All participants signed a written informed consent form before being interviewed and examined by a trained team.

## 3. Results

### 3.1. Sample Characteristics

The analytic sample comprised 227 medical students spanning all academic years of the programme. Mean age was 21 years (SD = 2), with female students representing 54.6% of the cohort. Total GAD-7 scores displayed moderate dispersion around the scale midpoint, with a distribution extending across the full response range. The proximity of central tendency values to commonly used screening thresholds reinforced the relevance of examining latent structure and measurement behaviour rather than relying on categorical classifications.

### 3.2. Factorial Structure

Three confirmatory factor models were estimated using WLSMV. The first corresponded to the original unidimensional structure of the GAD-7. Two alternative models were subsequently specified by progressively removing items with standardised factor loadings below 0.60 and 0.70, respectively. These reduced specifications were examined as sensitivity analyses to mirror common item-pruning approaches in the student-sample literature but were not treated as theoretically motivated refinements of the construct. Fit indices for all tested models are summarised in Table 1.

Across models, incremental fit indices (CFI, TLI) consistently approached or exceeded conventional reference values, whereas absolute fit indicators displayed greater variability. RMSEA values remained within acceptable ranges for Models 1 and 2, while Model 3 exhibited a marked increase, accompanied by an elevated χ^2^/df ratio. Progressive item removal produced marginal improvements in incremental fit at the expense of content coverage and absolute model behaviour, suggesting limited empirical justification for departing from the original specification.

### 3.3. Convergent Validity and Internal Consistency

Estimates of convergent validity and reliability are reported in Table 2. Average variance extracted increased monotonically across the three models, reflecting the mechanical effect of item removal on shared variance. Reliability coefficients remained stable across specifications.

The AVE value for the original model exceeded the recommended threshold of 0.50, indicating that the latent factor accounted for a substantial proportion of item variance. The absence of meaningful variation in α and ω across models suggested that internal consistency was not materially affected by item exclusion, reinforcing the interpretative trade-off between statistical refinement and content coverage.

### 3.4. Measurement Invariance Across Sex

Measurement invariance of the original unidimensional model was examined across sex using a hierarchical sequence of increasingly constrained models. Fit indices and model comparisons are presented in Table 3.

Configural equivalence was supported, indicating comparable factor structure across groups. Threshold and scalar constraints were retained without meaningful deterioration in incremental fit indices. The metric model displayed a slight increase in SRMR beyond commonly referenced cut-offs, while ΔCFI and ΔRMSEA remained within recommended bounds. Residual invariance imposed the most restrictive assumptions, with stable incremental indices but cumulative increases in absolute misfit.

## 4. Discussion

The psychometric behaviour of the GAD-7 within this cohort reflects a pattern recurrently observed in academically demanding populations, where anxiety-related symptom endorsement clusters near screening thresholds without necessarily crystallising into distinct factorial subdivisions. The unidimensional specification retained structural coherence under WLSMV estimation, even as alternative models produced marginal improvements in selected indices through progressive item removal. Similar trade-offs between parsimony and statistical refinement have been documented in university samples from South Korea, China and the United States, where item pruning yielded modest gains in incremental fit while attenuating content breadth [2,11,29].

The behaviour of absolute fit indices deserves particular attention. RMSEA values for the original model approached, but did not exceed, conventional upper bounds, a pattern frequently reported in short scales with limited degrees of freedom and ordinal indicators [25]. The sharper deterioration observed in the most restrictive alternative model suggests that elevated RMSEA in such contexts may reflect model underspecification induced by item removal rather than latent multidimensionality. Comparable dynamics have been noted in validations conducted among medical trainees in Asia and Europe, where attempts to enforce higher loading thresholds resulted in inflated χ^2^/df ratios and reduced model stability [2,10].

Convergent validity estimates further contextualise these findings. The AVE value obtained for the original model exceeded the 0.50 criterion proposed by Fornell and Larcker [26], aligning with or surpassing values indirectly inferred in prior studies relying on correlational approaches with instruments such as the PHQ-9, DASS-21 or HADS [4,30]. The monotonic increase in AVE across reduced models illustrates a mechanical consequence of item exclusion rather than a substantive enhancement of construct representation. This pattern reinforces concerns raised in the psychometric literature regarding the overinterpretation of incremental validity gains achieved through content narrowing, particularly in screening instruments intended to capture heterogeneous symptom expressions.

Reliability coefficients remained consistently high across all tested specifications, with α and ω values comparable to those reported in medical student samples from China (α = 0.91), South Korea (α = 0.93) and Spain (α = 0.94) [2,8,29]. While such estimates are often presented as unequivocal strengths, their stability across progressively restricted models suggests that internal consistency alone provides limited insight into the adequacy of latent representation. In homogenous academic samples, elevated reliability may coexist with restricted construct variance, underscoring the need to interpret these coefficients alongside factorial behaviour rather than in isolation.

Measurement invariance analyses introduce a further layer of complexity. Configural and scalar equivalence across sex were supported, permitting latent mean comparisons without systematic bias attributable to item thresholds. This pattern mirrors findings reported in primary care and university populations in Europe and North America, where scalar invariance was achieved despite partial instability at the metric level [10,11]. The observed increase in SRMR under metric constraints echoes these reports, suggesting that factor loadings may not operate identically across groups even when overall construct alignment is preserved. Given documented sex differences in anxiety symptom expression, this partial non-equivalence raises questions about whether strict metric invariance is a realistic expectation for constructs shaped by both biological and sociocultural processes.

Situated within the broader literature, these results contribute to an emerging view of the GAD-7 as a psychometrically resilient instrument whose behaviour is nonetheless sensitive to contextual embedding. In medical education, anxiety-related symptoms may oscillate between adaptive vigilance and maladaptive distress, challenging the assumption that screening tools function uniformly across clinical and academic environments. Studies reporting higher mean scores among medical students frequently rely on fixed cut-off points derived from primary care samples, an approach that may obscure latent structure dynamics and inflate prevalence estimates [1]. The present findings align with calls to re-examine how anxiety is operationalised when institutional design, rather than individual psychopathology alone, shapes symptom salience.

Several methodological considerations frame these interpretations. The non-probabilistic sampling strategy constrains generalisability, although such designs are common in psychometric investigations prioritising latent structure over population inference [15]. The exclusive focus on medical students limits extrapolation to other academic disciplines, where stressors and coping demands may differ qualitatively. Additionally, self-administration via digital platforms may modulate response patterns, particularly in cultures where mental health stigma influences disclosure.

Rather than resolving debates surrounding the internal structure of the GAD-7, these findings situate its measurement properties within a specific educational ecology. The persistence of a unidimensional configuration alongside minor tensions in fit and invariance indices suggests that anxiety, as captured by the GAD-7 in medical students, remains a fluid construct, responsive to contextual pressures and analytic choices alike. This openness invites further comparative work across disciplines, cultures and stages of professional training, where shifts in latent structure may illuminate not only measurement performance but the evolving phenomenology of anxiety itself.

### Methodological Considerations and Limitations

Several methodological features delimit the scope within which the present results can be interpreted. The use of a non-probabilistic quota sampling strategy restricts population-level inference, particularly with respect to prevalence estimation or normative score interpretation. While such designs are common and often appropriate in psychometric research focused on latent structure and measurement behaviour, they necessarily prioritise internal coherence over representativeness. As a result, the observed factorial and invariance patterns should be read as context-dependent rather than as universal properties of the instrument.

The exclusive focus on medical students introduces an additional layer of specificity. Medical training environments concentrate academic pressure, evaluative density and anticipatory professional stress in ways that may not generalise to other university disciplines. Anxiety-related symptoms in this setting may function partly as adaptive responses to institutional demands, complicating their alignment with constructs originally operationalised in primary care or community samples. This contextual embedding may help explain the proximity of central tendency scores to conventional screening thresholds and the relative stability of unidimensional solutions despite recurrent reports of alternative structures in broader student populations.

Data collection relied on self-administered digital questionnaires, a modality that may influence response patterns through reduced interviewer presence and perceived anonymity. While such conditions can facilitate disclosure, they may also amplify transient affective states linked to academic workload cycles. The absence of concurrent clinical interviews or external anxiety measures limits triangulation of latent scores with diagnostic or behavioural indicators and precluded examination of convergent and discriminant validity against theoretically related constructs (e.g., depression, perceived stress, burnout), constraining interpretation to measurement performance rather than clinical classification.

From an analytic perspective, the use of WLSMV estimation and polychoric correlations was appropriate for ordinal indicators exhibiting non-normal distributions. Nonetheless, fit indices such as RMSEA are known to behave unpredictably in models with few degrees of freedom, particularly when item counts are low. The modest tensions observed between absolute and incremental fit indicators may therefore reflect statistical artefacts as much as substantive model misspecification. Similarly, partial instability under metric invariance constraints suggests that strict equality of factor loadings across sex may be an unrealistic expectation for constructs shaped by both biological sensitivity and sociocultural expression norms. Given the study’s primary aim and sample size, we did not pursue partial invariance or item-level differential item functioning analyses; future research could use larger multi-institution samples to identify which indicators contribute most to loading non-equivalence.

Finally, the cross-sectional nature of the data precludes examination of temporal stability or sensitivity to change, dimensions that are especially relevant in academic environments characterised by cyclical stress exposure. Longitudinal designs could clarify whether the latent structure of the GAD-7 remains stable across training stages or fluctuates in response to institutional transitions, such as entry into clinical rotations or high-stakes examination periods.

Rather than undermining the present findings, these considerations delineate the conditions under which the GAD-7 appears to operate coherently within medical education. They also highlight directions for future work in which psychometric evaluation is embedded within a broader examination of how anxiety is produced, regulated and measured in professional training contexts.

## 5. Conclusions

Across a cohort of medical students spanning all academic years, the Spanish GAD-7 showed a structurally stable unidimensional configuration under ordinal CFA and high internal consistency. Scalar invariance across sex supports meaningful latent comparisons, while the less stable metric constraints suggest small differences in item salience between groups. In medical education—where anxiety-related symptoms may reflect both adaptive vigilance and clinically relevant distress—the GAD-7 appears suitable as a brief screening measure, provided interpretations remain context-sensitive and avoid population-level inference given the non-probabilistic design and the absence of external validity indicators in the present study.

## Figures and Tables

**Table 1:** Confirmatory factor analysis fit indices for tested GAD-7 models.

Model	χ^2^	df	*p*	χ^2^/df	RMSEA	RMSEA 90% CI (LCI–UCI)	*p* (RMSEA ≤ 0.05)	SRMR	CFI	TLI
**Model 1**	31.91	14	0.004	2.28	0.075	0.041–0.110	0.105	0.033	0.993	0.990
**Model 2**	19.77	9	0.019	2.20	0.073	0.028–0.117	0.170	0.026	0.996	0.993
**Model 3**	18.82	5	0.002	3.76	0.111	0.060–0.166	0.026	0.027	0.994	0.988

Note. Model 1 = original unidimensional model estimated with WLSMV. Model 2 = alternative model excluding items with standardised loadings < 0.60. Model 3 = alternative model excluding items with standardised loadings < 0.70.

**Table 2:** Convergent validity and reliability estimate across tested models.

Factor	AVE	Cronbach’s α	McDonald’s ω
**Model 1**	0.63	0.92	0.90
**Model 2**	0.67	0.92	0.90
**Model 3**	0.72	0.92	0.90

Note. AVE = average variance extracted.

**Table 3:** Measurement invariance testing across sex for the original GAD-7 model.

Model	χ^2^	df	*p*	χ^2^/df	RMSEA	SRMR	CFI	TLI	ΔCFI	ΔTLI	ΔRMSEA	ΔSRMR
**Configural**	47.39	28	0.012	1.69	0.078	0.042	0.994	0.990	—	—	—	—
**Thresholds**	46.46	35	0.093	1.33	0.054	0.042	0.996	0.995	0.002	0.005	−0.024	0.000
**Metric**	68.33	41	0.005	1.67	0.077	0.064	0.991	0.991	−0.005	−0.004	0.023	0.022
**Scalar**	78.59	47	0.003	1.67	0.077	0.064	0.990	0.991	−0.001	0.000	0.000	0.000
**Residual**	87.86	54	0.002	1.63	0.075	0.064	0.989	0.991	−0.001	0.000	−0.002	0.000

Note. Configural = no equality constraints. Thresholds = equal item thresholds. Metric = equal factor loadings. Scalar = equal factor loadings and thresholds (i.e., strong invariance). Residual = equal residual variances. Δ values represent change relative to the preceding (less constrained) model.

## Data Availability

The data presented in this study are available on reasonable request from the corresponding authors. The data are not publicly available due to ethical and privacy restrictions.

## Abbreviations

The following abbreviations are used in this manuscript:

Abbreviation	Meaning
APC	Article Processing Charge
AVE	Average Variance Extracted
CFA	Confirmatory Factor Analysis
CFI	Comparative Fit Index
CI	Confidence Interval
df	Degrees of Freedom
IRB	Institutional Review Board
RMSEA	Root Mean Square Error of Approximation
SD	Standard Deviation
SRMR	Standardised Root Mean Square Residual
TLI	Tucker–Lewis Index
WLSMV	Weighted Least Squares Mean- and Variance-Adjusted Estimator
α	Cronbach’s Alpha
ω	McDonald’s Omega

## References

[1] Quek T.T.-C., Tam W.W.-S., Tran B.X., Zhang M., Zhang Z., Ho C.S.-H., Ho R.C.-M. (2019). The Global Prevalence of Anxiety Among Medical Students: A Meta-Analysis. Int. J. Environ. Res. Public Health.

[2] Lee B., Kim Y.E. (2019). The psychometric properties of the Generalized Anxiety Disorder scale (GAD-7) among Korean university students. Psychiatry Clin. Psychopharmacol..

[3] Löwe B., Decker O., Müller S., Brähler E., Schellberg D., Herzog W., Herzberg P.Y. (2008). Validation and Standardization of the Generalized Anxiety Disorder Screener (GAD-7) in the General Population. Med. Care.

[4] Sousa T.V., Viveiros V., Chai M.V., Vicente F.L., Jesus G., Carnot M.J., Gordo A.C., Ferreira P.L. (2015). Reliability and validity of the Portuguese version of the Generalized Anxiety Disorder (GAD-7) scale. Health Qual. Life Outcomes.

[5] Spitzer R.L., Kroenke K., Williams J.B.W., Löwe B. (2006). A Brief Measure for Assessing Generalized Anxiety Disorder: The GAD-7. Arch. Intern. Med..

[6] Dhira T.A., Rahman M.A., Sarker A.R., Mehareen J. (2021). Validity and reliability of the Generalized Anxiety Disorder-7 (GAD-7) among university students of Bangladesh. PLoS ONE.

[7] Alghadir A., Manzar M.D., Anwer S., Albougami A., Salahuddin M. (2020). Psychometric Properties of the Generalized Anxiety Disorder Scale Among Saudi University Male Students. Neuropsychiatr. Dis. Treat..

[8] Garcia-Campayo J., Zamorano E., Ruiz M.A., Pardo A., Perez-Paramo M., Lopez-Gomez V., Freire O., Rejas J. (2010). Cultural adaptation into Spanish of the generalized anxiety disorder-7 (GAD-7) scale as a screening tool. Health Qual. Life Outcomes.

[9] Sun J., Liang K., Chi X., Chen S. (2021). Psychometric Properties of the Generalized Anxiety Disorder Scale-7 Item (GAD-7) in a Large Sample of Chinese Adolescents. Healthcare.

[10] Moreno E., Muñoz-Navarro R., Medrano L.A., González-Blanch C., Ruiz-Rodríguez P., Limonero J.T., Moretti L.S., Cano-Vindel A., Moriana J.A. (2019). Factorial invariance of a computerized version of the GAD-7 across various demographic groups and over time in primary care patients. J. Affect. Disord..

[11] Sriken J., Johnsen S.T., Smith H., Sherman M.F., Erford B.T. (2022). Testing the Factorial Validity and Measurement Invariance of College Student Scores on the Generalized Anxiety Disorder (GAD-7) Scale Across Gender and Race. Meas. Eval. Couns. Dev..

[12] Villarreal-Zegarra D., Paredes-Angeles R., Mayo-Puchoc N., Arenas-Minaya E., Huarcaya-Victoria J., Copez-Lonzoy A. (2024). Psychometric properties of the GAD-7 (General Anxiety Disorder-7): A cross-sectional study of the Peruvian general population. BMC Psychology.

[13] Franco-Jimenez R., Núñez-Magallanes A. (2022). Propiedades psicométricas del GAD-7, GAD-2 y GAD-Mini en universitarios peruanos. Propósitos Y Represent..

[14] Moreno-Montero E., Moreta-Herrera R., Rodas J.A., Oriol-Granado X., Puerta-Cortés D.X., Ferrufino-Borja D., Gismondi Diaz R., Lobos Rivera M.E., Samaniego-Pinho A., Buenahora-Bernal M. (2025). Cross-cultural measurement equivalence of the seven item general anxiety disorder scale (GAD-7) in college students of six countries of Latin America. J. Affect. Disord..

[15] Boateng G.O., Neilands T.B., Frongillo E.A., Melgar-Quiñonez H.R., Young S.L. (2018). Best Practices for Developing and Validating Scales for Health, Social, and Behavioral Research: A Primer. Front. Public Health.

[16] Worthington R.L., Whittaker T.A. (2006). Scale Development Research: A Content Analysis and Recommendations for Best Practices. Couns. Psychol..

[17] Dunn T.J., Baguley T., Brunsden V. (2014). From alpha to omega: A practical solution to the pervasive problem of internal consistency estimation. Br. J. Psychol..

[18] Beaton D.E., Bombardier C., Guillemin F., Ferraz M.B. (2000). Guidelines for the Process of Cross-Cultural Adaptation of Self-Report Measures. Spine.

[19] Hernan M.-C., Cristina M.M. (1994). Estratificación social y biología humana. Acta Científica Venez..

[20] Finch J.F., West S.G., MacKinnon D.P. (1997). Effects of sample size and nonnormality on the estimation of mediated effects in latent variable models. Struct. Equ. Model. Multidiscip. J..

[21] Flora D.B., Curran P.J. (2004). An Empirical Evaluation of Alternative Methods of Estimation for Confirmatory Factor Analysis With Ordinal Data. Psychol. Methods.

[22] Li C.-H. (2016). Confirmatory factor analysis with ordinal data: Comparing robust maximum likelihood and diagonally weighted least squares. Behav. Res. Methods.

[23] Muthén B., Asparouhov T. (2012). Bayesian structural equation modeling: A more flexible representation of substantive theory. Psychol. Methods.

[24] Hu L., Bentler P.M. (1999). Cutoff criteria for fit indexes in covariance structure analysis: Conventional criteria versus new alternatives. Struct. Equ. Model. Multidiscip. J..

[25] Marsh H.W., Hau K.-T., Wen Z. (2004). In Search of Golden Rules: Comment on Hypothesis-Testing Approaches to Setting Cutoff Values for Fit Indexes and Dangers in Overgeneralizing Hu and Bentler’s (1999) Findings. Struct. Equ. Model. Multidiscip. J..

[26] Fornell C., Larcker D.F. (1981). Evaluating Structural Equation Models with Unobservable Variables and Measurement Error. J. Mark. Res..

[27] Meredith W. (1993). Measurement Invariance, Factor Analysis and Factorial Invariance. Psychometrika.

[28] Chen F.F. (2007). Sensitivity of Goodness of Fit Indexes to Lack of Measurement Invariance. Struct. Equ. Model. Multidiscip. J..

[29] Zhang C., Wang T., Zeng P., Zhao M., Zhang G., Zhai S., Meng L., Wang Y., Liu D. (2021). Reliability, Validity, and Measurement Invariance of the General Anxiety Disorder Scale Among Chinese Medical University Students. Front. Psychiatry.

[30] Omani-Samani R., Maroufizadeh S., Ghaheri A., Navid B. (2018). Generalized Anxiety Disorder-7 (GAD-7) in people with infertility: A reliability and validity study. Middle East Fertil. Soc. J..

